# Expression profile and prognostic value of SFN in human ovarian cancer

**DOI:** 10.1042/BSR20190100

**Published:** 2019-05-02

**Authors:** Yi Hu, Qing Zeng, Chenxi Li, Ying Xie

**Affiliations:** 1 Department of Gynecology and Obstetrics, The Central Hospital of Wuhan, Tongji Medical College, Huazhong University of Science and Technology, Wuhan, China; 2 Department of Network Medicine, The Central Hospital of Wuhan, Tongji Medical College, Huazhong University of Science and Technology, Wuhan, China; 3 Department of Oral and Maxillofacial Surgery, Laboratory for Tumor Genetics and Regenerative Medicine, The Head and Neurocenter, University Medical Center Hamburg-Eppendorf (UKE), Hamburg 20246, Germany

**Keywords:** bioinformatic analysis, expression, Ovarian cancer, prognosis, Stratifin

## Abstract

Ovarian cancer is a highly lethal cancer in females. Therefore, it is necessary to explore effective biomarkers for the diagnosis and prognosis of the disease. Stratifin (SFN) is a cell cycle checkpoint protein that has been reported to be involved in oncogenesis. Our studies detected the expression of SFN in ovarian cancer by Oncomine, Human Protein Atlas database and ULCAN database. Meanwhile, we found its coexpression gene by cBioPortal online tool and validated their expression in different ovarian cancer cells by western blot and reverse transcription quantitative PCR. Then, we also investigated their prognostic values via the Kaplan–Meier plotter database in different subtypes of ovarian cancer patients. The results demonstrated that SFN was found to be increased in ten various ovarian cancer datasets, compared with healthy tissues. Additionally, up-regulation of SFN expression is associated with age and cancer grades. The higher expression of SFN in all patients with ovarian cancers is significantly correlated with worse postprogression survival. In addition, high SFN expression is associated with significantly worse overall survival in patients who received chemotherapy contains gemcitabine, taxol, taxol+platin, paclitaxel and avastin. In human ovarian carcinoma SKOV3 and A2780 cells, the expression of SFN and its coexpression gene MICB were also increased at protein and mRNA levels compared with the normal ovarian epithelial cells. Based on above results, overexpression of SFN was correlated with the prognosis in ovarian cancer. The present study might be useful for better understanding the clinical significance of SFN mRNA.

## Introduction

Ovarian cancer, a highly aggressive and lethal cancer, is in seventh place amongst all cancers in women worldwide in terms of occurrence [1]. Some statistical data displayed that up to 225,000 women were diagnosed with ovarian cancer and approximately 150,000 people die of this disease every year [2,3]. Although the development of the early diagnosis and treatment in ovarian cancer has been accomplished in recent decades, this disease is still a severe global burden and attracts more attention from the public. Early diagnosis of biomarkers and more accurate prognosis can optimize the efficiency of current ovarian cancer therapy and provide potential molecular markers for targetted treatment. Therefore, it is necessary that screening and identification of high efficiency molecular biomarkers regarding on ovarian cancer.

Stratifin (SFN) is a cell cycle checkpoint protein and a regulator of mitotic translation. To response the DNA damage, SFN often plays a role in inhibiting DNA errors during mitosis [4,5]. Generally, SFN is expressed at low levels on the nervous and reproductive systems, including in the cortex, retina, placenta, and ovary [6]. Nevertheless, SFN has been reported to be a novel biomarker in various cancers. For example, some studies found that SFN could facilitate progression and development of lung adenocarcinoma at an early stage [7]. Besides, combined with OCIAD2, immunocytochemical staining for SFN could also enhance diagnostic sensitivity for lung cancers [8]. For breast cancer, it is determined that the biology of SFN is important in cellular movement and is contingent on breast cancer subtype [9]. Meanwhile, Umbricht et al. also demonstrated that loss of expression of SFN is an early event in breast cancer transformation [10]. As a prognostic marker, it was reported that reduced SFN expression can serve as an independent prognostic factor for poor survival in patients with esophageal squamous cell carcinoma [11]. However, there existed little studies focussing the role of SFN in ovarian cancer. And its potential molecular mechanism and prognostic value have not yet been explored and clarified in different subtypes of ovarian cancers.

In the current analysis, we investigated the expression and prognosis of SFN and its coexpression gene in ovarian cancer for the first time by using bioinformatics. Hoping that this research may contribute to understand the role of SFN in detection and prognosis of ovarian cancer and development of therapeutic drugs for treatment of ovarian cancer.

## Materials and methods

### Ethics statement

All the datasets were acquired from the publishing literature and met with the Declaration of Helsinki. And we confirm that no human or animal specimens were used in our studies.

### Oncomine database

Oncomine database, a cancer database bioinformatics tool and online data-mining platform, was used to explore the mRNA levels of SFN and MHC class I polypeptide-related sequence B (MICB) in ovarian cancer. We searched the database for the fold changes of SFN and MICB in ovarian cancer using the filters of differential analysis (cancer vs normal), cancer type (ovarian cancer), sample type (clinical specimen), data type (mRNA), and gene (SFN and MICB). Students’ *t* test was used to generate a *P* value. To obtain the most significant SFN and MICB probes, we set the following parameters: the cutoff of *P* value and fold change were defined as 0.01 and 1.5, respectively

### GEPIA database

Gene expression profiling interactive analysis (GEPIA) (http://gepia.cancer-pku.cn) database, is a newly developed interactive web server developed by Zefang T, Chenwei L, and Boxi K of Zhang Lab, Peking University, focussed on scrutinizing the RNA sequencing expression data of 9736 tumors and 8587 normal samples from the TCGA and the GTEx projects, using a standard processing pipeline. In the present study, we used GEPIA to validate the correlation between the expression levels of candidate genes.

### Human Protein Atlas database

Human Protein Atlas (http://www.proteinatlas.org) is a Swedish-based program initiated in 2003 with the aim to map all the human proteins in cells, tissues, and organs using integration of various omics technologies. In our study, the Human Protein Atlas was used for immunohistochemistry validation of several candidate genes.

### UALCAN database

The UALCAN web portal has an important feature that aids querying based on the gene class. Meanwhile, it is a userfriendly, interactive web resource for analyzing cancer transcriptome data. It is built on PERL-CGI with high quality graphics using javascript and CSS. UALCAN is designed to, (1) provide easy access to publicly available cancer transcriptome data (TCGA and MET500 transcriptome sequencing), (2) allow users to identify biomarkers or to perform *in silico* validation of potential genes of interest, (3) provide publication quality graphs and plots depicting gene expression and patient survival information based on gene expression, and (4) evaluate gene expression in molecular subtypes of breast and prostate cancer. Using UALCAN database, we analyzed the expression profiles of SFN in normal and ovarian serous cystadenocarcinoma samples based on clinicopathologic parameters, such as cancer stage, age, race, and tumor grade.

### cBioPortal database

The cBioPortal for Cancer Genomics was originally developed at Memorial Sloan Kettering Cancer Center, which contained both sequencing and pathological data on 30 different cancers. The ovarian cancer (TCGA, ovarian serous cystadenocarcinoma; provisional) dataset including data from 617 samples with pathology reports was selected for further analyses of SFN. According to the cBioPortal’s online tool, the genomic profiles included the genetic alterations, survival curve, and correlations of SFN.

### Cell culture

Human ovarian carcinoma SKOV3 and A2780 cells were bought from ATCC (Manassas VA, U.S.A.). Normal ovarian epithelial IOSE80 cells were purchased from MssBio Co., Ltd. (Guangzhou, China). SKOV3 and A2780 cells were cultured in 80% RPMI 1640 medium (Sigma–Aldrich, St Louis, MO, U.S.A.), supplemented with 10% FBS (Gibco, U.S.A.), 10% horse serum (Gibco), 100 units/ml penicillin, and 100 μg/ml streptomycin. The IOSE80 cells were cultured in RPMI-1640 medium (Sigma–Aldrich) supplemented with 10% FBS. All the cell lines were grown in a (5% CO_2_) humidified incubator at 37°C and passaged every 3–4 days.

### Reverse transcription quantitative PCR

Total RNA was prepared from all cell lines using an RNeasy Mini Plus Kit (Qiagen), and its quality was evaluated using an Agilent 2100 Bioanalyzer (Agilent Technologies, Waldbronn, Germany). To detect the expression of mRNA of SFN gene, reverse transcription quantitative PCR (RT-qPCR) was conducted on a 7300 PCR system (Thermo Fisher Scientific).

The primers were as follows:

SFN forward; 5-TCCACTACGAGATCGCCAACAG-3, and reverse; 5-GTGTCAGGTTGTCTCGCAGCA-3;

MICB forward 5′-CGGGATCCCACAGTCTTCGTTACAAC-3′,

and reverse 5′-CGGAATTCCTATGTCACGGTGATGTTGC-3;

GAPDH forward: 5′-CGCTAACATCAAATGGGGTG-3′ and reverse 5′-TTGCTGACAATCTTGAGGGAG-3′.

PCR amplified conditions: 40 cycles of denaturation at 95°C for 15 s, annealing at 56°C for 20 s, and extension at 72°C for 45 s.

The relative expression levels were calculated by the 2^−ΔΔ*C*^_T_ method. The mRNA levels of the target gene were normalized to the levels of glyceraldehyde-3-phosphate dehydrogenase (GAPDH). GAPDH was used as the endogenous ‘housekeeping’ control gene for these analyses

### Western blotting

Total proteins were prepared on ice using M-PER for cultured cells. Cultured cells were collected and cell lysates were prepared in the lysis buffer (Beyotime). The cell lysates were centrifuged at 2000 rpm for 5 min at 4°C collect the supernatants. Then the total protein concentrations in the supernatants were measured by using the bicinchoninic acid protein assay kit (Aspen, Wuhan, China), protein samples (30 μg) were submitted to electrophoresis on 10% SDS-polyacrylamide gel (Aspen) and transferred to polyvinylidene fluoride (PVDF) membrane (Millipore, Billerica, MA, U.S.A.). The membranes were probed with specific antibodies against SFN (1:1000, Cell Signaling) and GAPDH (1:5000, Cell Signaling). GAPDH was adopted as an internal control. Secondary antibody were detected with enhanced chemiluminescence reagents (Thermo Scientific, Darmstadt, Germany) according to the manufacturer’s instructions. Finally, the immunoreactions were detected by enhanced chemiluminescence (Aspen) and visualized by AlphaEaseFC software for image analysis.

### Statistical analyses

Data were analyzed in SPSS 19.0 software (IBM Corporation, Armonk, NY, U.S.A.). The data were expressed as the mean ± S.E.M. Statistical significance between groups was analyzed by using a one-way ANOVA. A value of *P*<0.05 was accepted as statistically significant.

## Results

### The expression level of SFN mRNA in human ovarian cancer

**Figure 1:**
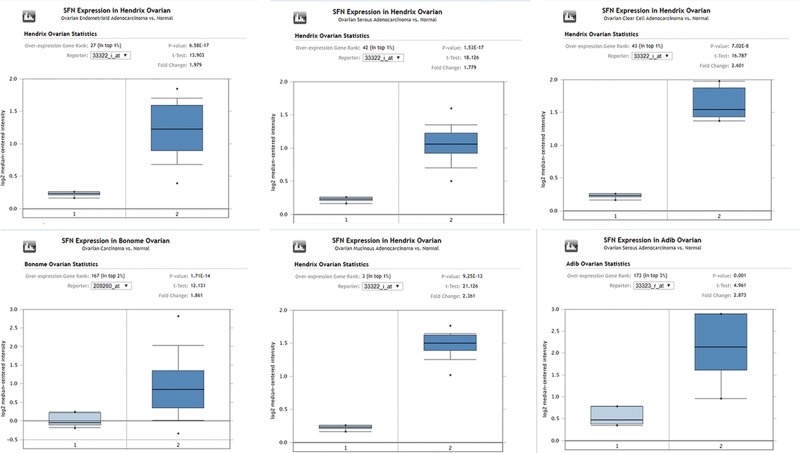
The expression level of SFN mRNA in human different types of ovarian cancer (ONCOMINE)

**Figure 2:**
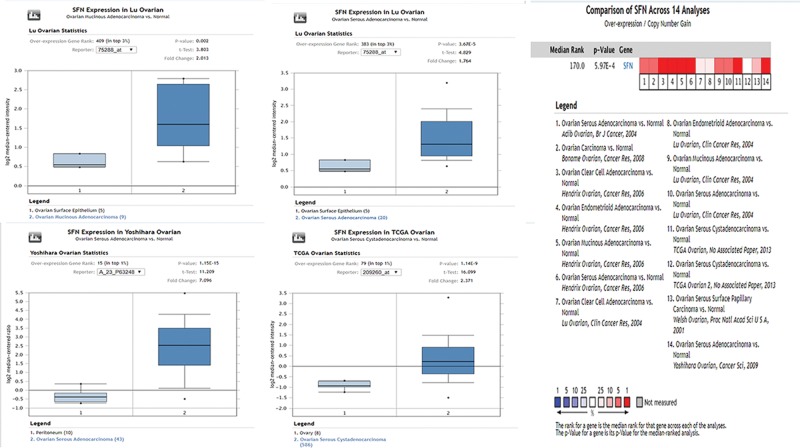
The expression level of SFN mRNA in human different types of ovarian cancer and meta-analysis (ONCOMINE)

As shown in Figure 1, we exhibited the expression levels of SFN in different kinds of ovarian cancer with those in control samples by exploring the Oncomine database. SFN was found to be increased in ten various ovarian cancer datasets, compared with healthy tissues (*P* value < 0.01 and fold change > 1.5) including ovarian mucinous adenocarcinoma [12,13], ovarian clear cell adenocarcinoma [12], ovarian carcinoma [14], ovarian serous adenocarcinoma [12,13,15,16], ovarian serous cystadenocarcinoma (from TCGA database), and ovarian endometrioid adenocarcinoma [12]. The comparisons of mRNA levels of SFN in ovarian cancer and healthy samples in each individual dataset were performed by using the Student’s *t* test (Figures 1 and 2).

### The correlation between the expression levels of SFN and the clinicopathological parameters of ovarian cancer patients

**Figure 3:**
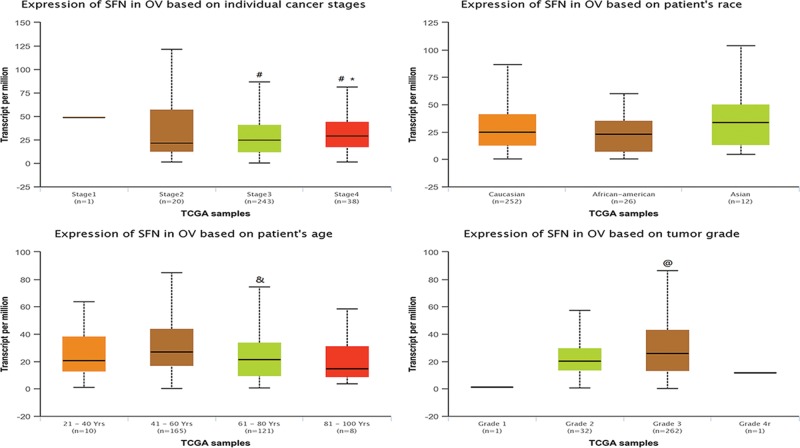
Association between SFN gene expression and clinical pathological parameters in patients with ovarian cancer (ULACAN) &*P*: compared with the age (41–60 years) group; @*P*: compared with the grade 2 group.

Using UALCAN database, we analyzed the expression profiles of SFN in normal and ovarian serous cystadenocarcinoma samples based on clinicopathologic parameters, such as cancer stage, age, race, and tumor grade. As shown in Figure 3, our analysis mining of the UALCAN database indicated that SFN was down-regulated in age (61–80 years) group compared with age (41–60 years) group (*P*<0.01). And for cancer grades, the expression level of SFN was higher in grade 3 compared with the grade 2 of ovarian serous cystadenocarcinoma (*P*<0.05). In terms of the cancer stage and patients’ race, there existed no significant difference in the expression of SFN amongst different groups.

**Figure 4:**
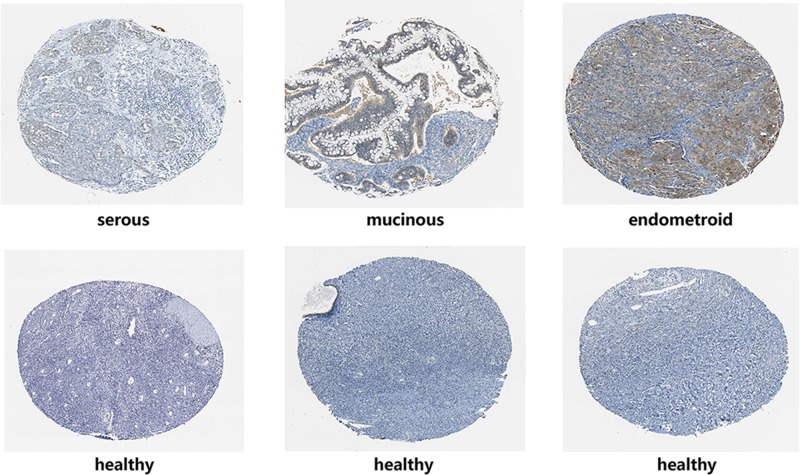
The IHC of SFN in ovarian cancer (HPA)

Subsequently, we searched the IHC pictures of different kinds of ovarian cancer in the HPA database. The IHC pictures displayed that the SFN seemed to show a positive intensity in ovarian cancers compared with those in normal samples, especially for ovarian mucinous adenocarcinoma and ovarian endometrioid adenocarcinoma, which further verified the results of Oncomine database (Figure 4).

### The Kaplan–Meier plotter in ovarian cancers

**Figure 5:**
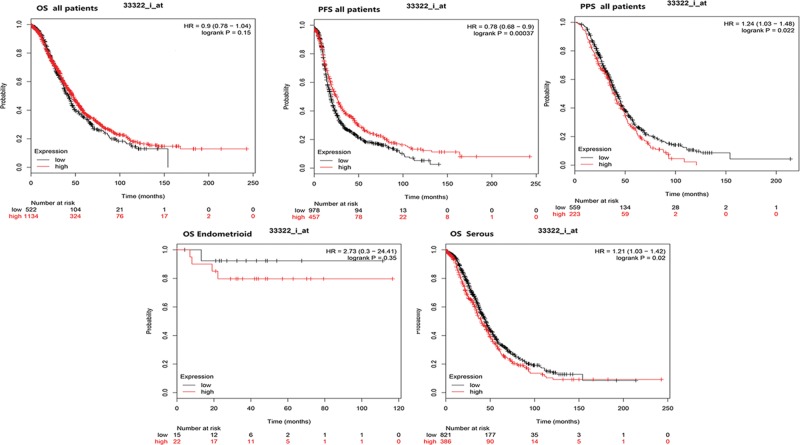
The prognostic value of SFN mRNA in ovarian cancer patients (Kaplan–Meier plotter)

**Figure 6:**
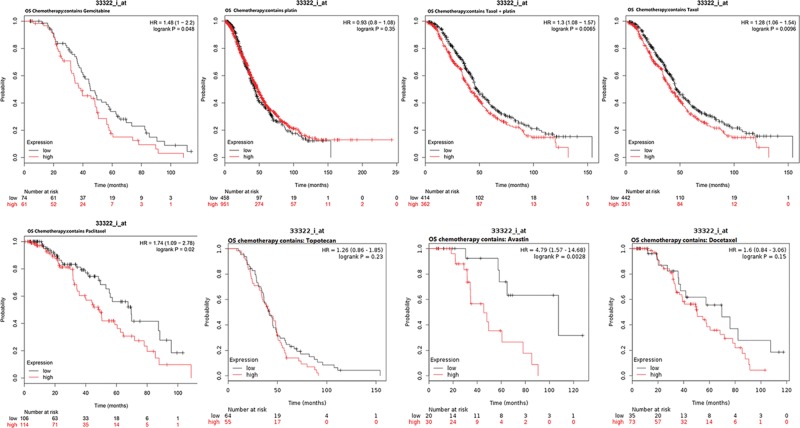
The prognostic value of SFN mRNA in ovarian cancer patients who received chemotherapy (Kaplan–Meier plotter)

The Kaplan–Meier plotter is capable to assess the effect of 54,675 genes on survival using 18,674 cancer samples. The prognostic values of SFN in various ovarian cancers were evaluated by Kaplan–Meier plotter. Although SFN expression was not significantly correlated with poor overall survival, the higher expression of SFN in all patients with ovarian cancers is significantly correlated with the better progression-free survival (PFS) and worse postprogression survival (PPS) *(P*<0.05). Further analysis found that higher mRNA levels of SFN was associated with poorer OS of ovarian serous adenocarcinoma patients (Figure 5). Considering that chemotherapy is a critical portion of ovarian cancer treatments. We detected the relationship between SFN expression and OS of ovarian cancer patients received different chemotherapy drug. As shown in Figures 5 and 6, high SFN expression is associated with significantly worse OS in patients who received chemotherapy contains gemcitabine, taxol, taxol+platin, paclitaxel and avastin (*P*<0.05). For chemotherapy contains platin, topotecan, and docetaxel, the expression level of SFN was not associated with the OS in all ovarian patients (Figure 6).

### Analysis of SFN’s alterations and coexpressed genes in ovarian cancer

**Figure 7:**
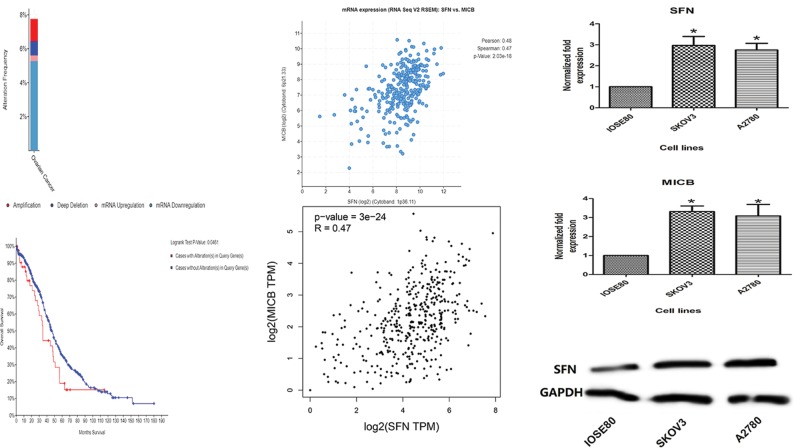
Analysis of SFN’s alterations and coexpressed genes in ovarian cancer (**A**) The alteration frequency of SFN in ovarian cancer. (**B**) The survival curve of SFN alteration in ovarian cancer. (**C**) Through CbioPortal tool, SFN is mainly correlated with MICB. (**D**) Data mining in GEPIA database confirmed the positive correlation of SFN and MICB expression. (**E**) Histogram of the difference between SFN expression levels in ovarian carcinoma cell of SKOV3, A2780, and normal ovarian epithelial cell lines. (**F**) Histogram of the difference between MICB expression levels in ovarian carcinoma cell of SKOV3, A2780, and normal ovarian epithelial cell lines. *Compared with the normal groups, *P*<0.05. (**G**) Western blot was performed to detect levels of SFN in ovarian carcinoma cell of SKOV3, A2780, and normal ovarian epithelial cell lines.

Based on cBioPortal online database for ovarian serous adenocarcinoma (TCGA, provisional), we explored the genetic alterations, correlations, and OS of SFN in Ovarian Serous Adenocarcinoma. In this database, SFN gene altered in 47 cases out of 606 cases (7.76%). In the top ten coexpression genes, we found that SFN was positively correlated with MICB, BCL3, TRIM47, SLC43A3, TAGLN2, OSTF1, KRT16, FAM111B, EBP, and NQO1 (Table 1). Through investigating patient with ovarian serous adenocarcinoma data in TCGA database using GEPIA database, the positive correlation between MICB and SFN was confirmed (Figure 7A–D). These data suggested that SFN could be associated with the MICB in ovarian cancer. In addition, by log-rank tests in the Kaplan–Meier plots, we also discovered that ovarian serous adenocarcinoma cases with alterations in SFN gene had worse OS (*P*=0.0461).

**Table 1:** Coexpression genes of SFN in ovarian cancer

Correlated gene	Cytoband	Pearson’s correlation	Spearman’s correlation	*P*-value	q-value
MICB	6p21.33	0.48	0.471758618	2.03E-18	1.17E-14
BCL3	19q13.32	0.47	0.471050665	2.32E-18	1.17E-14
TRIM47	17q25.1	0.46	0.467819049	4.23E-18	1.43E-14
SLC43A3	11q12.1	0.46	0.46776306	4.27E-18	1.43E-14
TAGLN2	1q23.2	0.45	0.462507123	1.12E-17	3.22E-14
OSTF1	9q21.13	0.51	0.45661125	3.23E-17	5.91E-14
KRT16	17q21.2	0.51	0.454688396	4.54E-17	7.03E-14
FAM111B	11q12.1	0.42	0.451002835	8.68E-17	1.17E-13
EBP	Xp11.23	0.39	0.446790324	1.80E-16	2.27E-13
NQO1	16q22.1	0.46	0.439833559	5.91E-16	5.94E-13

### Gene expression levels of SFN and its coexpressed gene in cancer cells

The RT-qPCR results displayed that the gene expression level of SFN and its coexpressed gene MICB in SKOV3 and A2780 cells was obviously higher than that of IOSE80 lines (Figure 7E,F). In western blotting, compared with the normal ovarian epithelial cells, the expression of SFN was significantly increased in SKOV3 and A2780 cells, which was also in accordance with the results of RT-qPCR (Figure 7G).

### MICB mRNA expression and prognosis in patients with ovarian cancer

**Figure 8:**
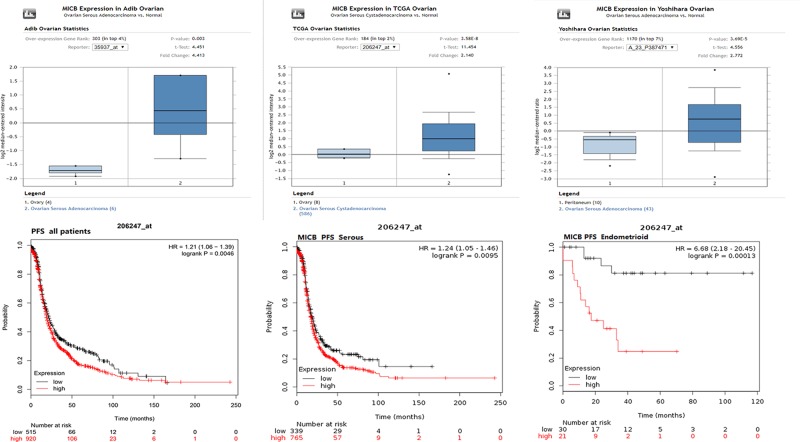
MICB mRNA expression and prognosis in patients with ovarian cancer (**A**) MICB mRNA expression analysis in Oncomine database. (**B**) The prognostic value of MICB in ovarian cancer by Kaplan–Meier plotter.

To verify the roles of MICB in ovarian cancer, the expression profiles of MICB were retrieved in the Oncomine database. The results of our analysis revealed that MICB expression had an obviously increased level in ovarian serous adenocarcinoma and ovarian serous cystadenocarcinoma compared with the normal tissues. Afterward, the prognostic value of MICB in ovarian cancer was examined via the Kaplan–Meier survival plots. The results indicated that a high expression of MICB mRNA was significantly associated with poor PFS in ovarian cancer patients, including ovarian serous carcinoma and ovarian endometrioid adenocarcinoma patients (Figure 8).

## Discussion

SFN, a cell cycle checkpoint protein, binds to translation and initiation factors and functions as a regulator of mitotic translation [7]. In response to DNA damage, this protein plays a role in preventing DNA errors during mitosis. Previous studies indicated that SFN participated in multiple kinds of tumor, including lung [17], renal [18], liver [19], and breast [20,21] cancer. Especially for lung cancer, several studies demonstrated that SFN played as a novel oncogene, stimulating the tumor initiation, and progression of lung adenocarcinoma [8,17]. Furthermore, they found that SFN enhances receptor tyrosine kinases stabilization by aberrant ubiquitin-specific protease 8 (USPB) regulation in lung adenocarcinoma, implying that SFN could be an appropriate therapeutic target for lung adenocarcinoma than USP8 [17]. As for ovarian cancer, current research just indicated that SFN was one of highly overexpressed genes in ovarian cancer and normal ovarian epithelium and may involve in regulation network of ovarian cancer. However, the comprehensive analysis of SFN as a diagnostic marker of prognosis and its coexpression genes in ovarian cancer are still unrevealed.

Our current analysis was built-in multiple cancer databases, which were all based on TCGA database. Through Oncomine database, we confirmed that SFN was remarkably increased in ovarian mucinous adenocarcinoma, ovarian clear cell adenocarcinoma, ovarian serous adenocarcinoma, ovarian serous cystadenocarcinoma and ovarian endometrioid adenocarcinoma. Moreover, we investigated the correlation between the expression levels of SFN and the clinicopathological parameters of ovarian cancer patients based on different subtypes. UALCAN database indicated that SFN was down-regulated in age (61–80 years) group compared with age (41–60 years) group. And for cancer grades, the expression level of SFN was higher in grade 3 compared with the grade 2 of ovarian serous cystadenocarcinoma. Meanwhile, the IHC pictures displayed that the SFN seemed to show a positive intensity in ovarian cancers compared with those in normal samples, especially for ovarian mucinous adenocarcinoma and ovarian endometrioid adenocarcinoma, which further verified the results of Oncomine database. Then the prognostic values of SFN in various ovarian cancers were evaluated by Kaplan–Meier plotter. Although SFN expression was not significantly correlated with poor overall survival, the higher expression of SFN in all patients with ovarian cancers is remarkably correlated with the better PFS and worse PPS. Further analysis found that higher mRNA levels of SFN was associated with poor OS of ovarian serous adenocarcinoma patients.

Considering that chemotherapy and cytoreductive surgery remained the gold standards of ovarian cancer treatment [22,23], we focussed on the relationship between SFN expression and OS of ovarian cancer patients received different chemotherapy drug. As a result, high SFN expression is associated with significantly worse OS in patients who received chemotherapy contains gemcitabine, taxol, taxol+platin, paclitaxel, and avastin. Taxol and platin were widely used and recommended as a first-line drug in the treatment of ovarian cancer. Gemcitabine and avastin acquired more attention in recent year, and more and more advanced ovarian benefited from these two drugs [24,25]. In the view of the huge number of ovarian cancer patients, SFN could be a potential prognostic marker for ovarian patients who received chemotherapeutics.

Based on coexpression and correlation data from cBioPortal online database, it was determined that the alteration frequency of SFN in ovarian cancer is not high. And MICB was co-up-regulated with SFN in ovarian cancer, which was confirmed by the GEPIA database, suggesting that SFN could be associated with the MICB in ovarian cancer. MICB, a heavily glycosylated protein, is a ligand for the NKG2D type II receptor [26,27]. This protein is stress-induced and similar to MHC class I molecules; however, it does not associate with β-2-microglobulin or bind peptides. Because of its role in antigen binding and natural killer cell lectin-like receptor binding, MICB had been found to be related in several types of cancers, involving lung cancer cell lines [28] and leukemia [29]. In our studies, we also demonstrated that MICB was notable up-regulated in ovarian serous adenocarcinoma and ovarian serous cystadenocarcinoma compared with the normal tissues. In addition, the survival analysis revealed that a high expression of MICB mRNA was significantly associated with poor PFS in ovarian cancer patients, including ovarian serous carcinoma and ovarian endometrioid adenocarcinoma patients, which was also similar with SFN’ survival analysis in ovarian cancer. Combined with the above data, we could suppose that SFN transcript expression may modulate the initiation and development of ovarian cancer and influence its prognosis, which may associate with MICB expression.

However, there are some limitations in our study. First, we investigated the expressions and prognosis value of SFN and MICB in ovarian cancer from several public databases. However, we did not validate these findings by PCR or immunohistochemistry. Second, the upstream molecule on the roles of SFN in ovarian cancer lack of further exploration. It is valued to be dig and verified in the future.

## Conclusion

In summary, SFN and its coexpressed gene MICB were significantly increased in multiple types of ovarian cancer. Furthermore, high expression of SFN predicted poor PPS in ovarian patients high SFN expression is associated with significantly worse OS in patients who received chemotherapy contains gemcitabine, taxol, taxol+platin, paclitaxel, and avastin. Besides, high expression of MICB predicted worse PFS in both ovarian serous carcinoma and ovarian endometrioid adenocarcinoma patients. The present study might be useful for better understanding the clinical significance of SFN mRNA and provided a potential therapeutic target for ovarian cancer research in the future.

## Abbreviations

GAPDH: glyceraldehyde-3-phosphate dehydrogenase
GEPIA: gene expression profiling interactive analysis
MICB: MHC class I polypeptide-related sequence B
PFS: progression-free survival
PPS: postprogression survival
PVDF: polyvinylidene fluoride
qRT-PCR: reverse transcription quantitative PCR
SFN: stratifin
USPB: ubiquitin-specific protease 8

## References

[1] Yoneoka Y., Yoshida H., Ishikawa M., Shimizu H., Uehara T., Murakami T. (2019) Prognostic factors of synchronous endometrial and ovarian endometrioid carcinoma. J. Gynecol. Oncol. 30, e7 10.3802/jgo.2019.30.e7 30479091 PMC6304406

[2] Huang L.J., Deng X.F., Chang F., Wu X.L., Wu Y. and Diao Q.Z. (2018) Prognostic significance of programmed cell death ligand 1 expression in patients with ovarian carcinoma: a systematic review and meta-analysis. Medicine (Baltimore) 97, e12858 10.1097/MD.0000000000012858 30412078 PMC6221561

[3] Santin A.D., Zhan F., Bellone S., Palmieri M., Cane S., Bignotti E. (2004) Gene expression profiles in primary ovarian serous papillary tumors and normal ovarian epithelium: identification of candidate molecular markers for ovarian cancer diagnosis and therapy. Int. J. Cancer 112, 14–25 10.1002/ijc.20408 15305371

[4] Rizou M., Frangou E.A., Marineli F., Prakoura N., Zoidakis J., Gakiopoulou H. (2018) The family of 14-3-3 proteins and specifically 14-3-3σ are up-regulated during the development of renal pathologies. J. Cell. Mol. Med. 22, 4139–4149 10.1111/jcmm.13691 29956451 PMC6111864

[5] Kaplan A., Bueno M. and Fournier A.E. (2017) Extracellular functions of 14-3-3 adaptor proteins. Cell. Signal. 31, 26–30 10.1016/j.cellsig.2016.12.007 27993556

[6] Winter M., Lodygin D., Verdoodt B. and Hermeking H. (2016) Deletion of 14-3-3σ sensitizes mice to DMBA/TPA-induced papillomatosis. Oncotarget 7, 46862–46870 10.18632/oncotarget.10478 27409835 PMC5216908

[7] Shiba-Ishii A., Kim Y., Shiozawa T., Iyama S., Satomi K., Kano J. (2015) Stratifin accelerates progression of lung adenocarcinoma at an early stage. Mol. Cancer 14, 142 10.1186/s12943-015-0414-1 26223682 PMC4518688

[8] Itoguchi N., Nakagawa T., Murata Y., Li D., Shiba-Ishii A., Minami Y. (2015) Immunocytochemical staining for stratifin and OCIAD2 in bronchial washing specimens increases sensitivity for diagnosis of lung cancer. Cytopathology 26, 354–361 10.1111/cyt.12220 25376185

[9] Boudreau A., Tanner K., Wang D., Geyer F.C., Reis-Filho J.S. and Bissell M.J. (2013) 14-3-3σ stabilizes a complex of soluble actin and intermediate filament to enable breast tumor invasion. Proc. Natl Acad. Sci. U.S.A. 110, E3937–E3944 10.1073/pnas.1315022110 24067649 PMC3799319

[10] Umbricht C.B., Evron E., Gabrielson E., Ferguson A., Marks J. and Sukumar S. (2001) Hypermethylation of 14-3-3 sigma (stratifin) is an early event in breast cancer. Oncogene 20, 3348–3353 10.1038/sj.onc.1204438 11423985

[11] Ren H.Z., Pan G.Q., Wang J.S., Wen J.F., Wang K.S., Luo G.Q. (2010) Reduced stratifin expression can serve as an independent prognostic factor for poor survival in patients with esophageal squamous cell carcinoma. Dig. Dis. Sci. 55, 2552–2560 10.1007/s10620-009-1065-0 20108042

[12] Hendrix N.D., Wu R., Kuick R., Schwartz D.R., Fearon E.R. and Cho K.R. (2006) Fibroblast growth factor 9 has oncogenic activity and is a downstream target of Wnt signaling in ovarian endometrioid adenocarcinomas. Cancer Res 66, 1354–1362 10.1158/0008-5472.CAN-05-3694 16452189

[13] Lu K.H., Patterson A.P., Wang L., Marquez R.T., Atkinson E.N., Baggerly K.A. (2004) Selection of potential markers for epithelial ovarian cancer with gene expression arrays and recursive descent partition analysis. Clin. Cancer Res. 10, 3291–3300 10.1158/1078-0432.CCR-03-0409 15161682

[14] Bonome T., Levine D.A., Shih J., Randonovich M., Pise-Masison C.A., Bogomolniy F. (2008) A gene signature predicting for survival in suboptimally debulked patients with ovarian cancer. Cancer Res. 68, 5478–5486 10.1158/0008-5472.CAN-07-6595 18593951 PMC7039050

[15] Yoshihara K., Tajima A., Komata D., Yamamoto T., Kodama S., Fujiwara H. (2009) Gene expression profiling of advanced-stage serous ovarian cancers distinguishes novel subclasses and implicates ZEB2 in tumor progression and prognosis. Cancer Sci. 100, 1421–1428 10.1111/j.1349-7006.2009.01204.x 19486012 PMC11159497

[16] Adib T.R., Henderson S., Perrett C., Hewitt D., Bourmpoulia D., Ledermann J. (2004) Predicting biomarkers for ovarian cancer using gene-expression microarrays. Br. J. Cancer 90, 686–692 10.1038/sj.bjc.6601603 14760385 PMC2409606

[17] Kim Y., Shiba-Ishii A., Nakagawa T., Iemura S.I., Natsume T., Nakano N. (2018) Stratifin regulates stabilization of receptor tyrosine kinases via interaction with ubiquitin-specific protease 8 in lung adenocarcinoma. Oncogene 37, 5387–5402 10.1038/s41388-018-0342-9 29880877

[18] Suárez-Bonnet A., Lara-García A., Stoll A.L., Carvalho S. and Priestnall S.L. (2018) 14-3-3σ protein expression in canine renal cell carcinomas. Vet. Pathol. 55, 233–240 10.1177/0300985817738097 29145797

[19] Reis H., Pütter C., Megger D.A., Bracht T., Weber F., Hoffmann A.C. (2015) A structured proteomic approach identifies 14-3-3Sigma as a novel and reliable protein biomarker in panel based differential diagnostics of liver tumors. Biochim. Biophys. Acta 1854, 641–650 10.1016/j.bbapap.2014.10.024 25448011

[20] Ben-David U., Ha G., Khadka P., Jin X., Wong B., Franke L. (2016) The landscape of chromosomal aberrations in breast cancer mouse models reveals driver-specific routes to tumorigenesis. Nat. Commun. 7, 12160 10.1038/ncomms12160 27374210 PMC4932194

[21] Plant H.C., Kashyap A.S., Manton K.J., Hollier B.G., Hurst C.P., Stein S.R. (2014) Differential subcellular and extracellular localisations of proteins required for insulin-like growth factor- and extracellular matrix-induced signalling events in breast cancer progression. BMC Cancer 14, 627 10.1186/1471-2407-14-627 25167778 PMC4158058

[22] Andersen C.L., Liu M., Wang Z., Ye X. and Xiao S. (2018) Chemotherapeutic agent doxorubicin alters uterine gene expression in response to estrogen in ovariectomized CD-1 adult mice. Biol. Reprod. 10.1093/biolre/ioy259 30561525 PMC6483053

[23] Rodriguez-Freixinos V., Fariñas-Madrid L., Gil-Martin M., Barretina-Ginesta P., Romeo M., Villacampa G. (2018) Chemotherapy and PARP inhibitors in heavily pretreated BRCA1/2 mutation ovarian cancer (BMOC) patients. Gynecol. Oncol. 10.1016/j.ygyno.2018.11.036 30551885

[24] Komiyama S., Kugimiya T., Takeya C., Takahashi R. and Kubushiro K. (2018) Platinum-resistant recurrent ovarian cancer with long survival on bevacizumab and gemcitabine. J. Obstet. Gynaecol. Res. 44, 1330–1334 10.1111/jog.13664 29767464

[25] Pignata S., C C.S., Du Bois A, Harter P. and Heitz F. (2017) Treatment of recurrent ovarian cancer. Ann. Oncol. 28, viii51–51viii56 10.1093/annonc/mdx441 29232464

[26] Schmiedel D. and Mandelboim O. (2018) NKG2D ligands-critical targets for cancer immune escape and therapy. Front. Immunol. 9, 2040 10.3389/fimmu.2018.02040 30254634 PMC6141707

[27] Wongfieng W., Jumnainsong A., Chamgramol Y., Sripa B. and Leelayuwat C. (2017) 5′-UTR and 3′-UTR regulation of MICB Expression In Human Cancer Cells By Novel MicroRNAs. Genes (Basel) 8, 10.3390/genes8090213 28850101 PMC5615347

[28] Luo D., Dong X.W., Yan B., Liu M., Xue T.H., Liu H. (2018) MG132 selectively up-regulates MICB through the DNA damage response pathway in A549 cells. Mol. Med. Rep. 10.3892/mmr.2018.9676 PMC6297755 30483783

[29] Baek I.C., Shin D.H., Choi E.J., Kim H.J., Yoon J.H., Cho B.S. (2018) Association of MICA and MICB polymorphisms with the susceptibility of leukemia in Korean patients. Blood Cancer J. 8, 58 10.1038/s41408-018-0092-5 29895953 PMC5997647

